# BCR-UNet: Bi-directional ConvLSTM residual U-Net for retinal blood vessel segmentation

**DOI:** 10.3389/fpubh.2022.1056226

**Published:** 2022-11-22

**Authors:** Yugen Yi, Changlu Guo, Yangtao Hu, Wei Zhou, Wenle Wang

**Affiliations:** ^1^School of Software, Jiangxi Normal University, Nanchang, China; ^2^Yichun Economic and Technological Development Zone, Yichun, China; ^3^The 908th Hospital of Chinese People's Liberation Army Joint Logistic Support Force, Nanchang, China; ^4^College of Computer Science, Shenyang Aerospace University, Shenyang, China

**Keywords:** segmentation, retinal blood vessels, U-Net, residual convolution, Bi-directional ConvLSTM

## Abstract

**Background:**

High precision segmentation of retinal blood vessels from retinal images is a significant step for doctors to diagnose many diseases such as glaucoma and cardiovascular diseases. However, at the peripheral region of vessels, previous U-Net-based segmentation methods failed to significantly preserve the low-contrast tiny vessels.

**Methods:**

For solving this challenge, we propose a novel network model called Bi-directional ConvLSTM Residual U-Net (BCR-UNet), which takes full advantage of U-Net, Dropblock, Residual convolution and Bi-directional ConvLSTM (BConvLSTM). In this proposed BCR-UNet model, we propose a novel Structured Dropout Residual Block (SDRB) instead of using the original U-Net convolutional block, to construct our network skeleton for improving the robustness of the network. Furthermore, to improve the discriminative ability of the network and preserve more original semantic information of tiny vessels, we adopt BConvLSTM to integrate the feature maps captured from the first residual block and the last up-convolutional layer in a nonlinear manner.

**Results and discussion:**

We conduct experiments on four public retinal blood vessel datasets, and the results show that the proposed BCR-UNet can preserve more tiny blood vessels at the low-contrast peripheral regions, even outperforming previous state-of-the-art methods.

## Introduction

Retinal vascular features play an essential role in physicians' diagnosis of early ophthalmic and cardiovascular diseases, as these diseases lead to morphological changes in retinal blood vessels. A typical example is diabetic retinopathy (DR), a retinal disease that is one of the leading causes of blindness and requires special attention if retinal vasodilation is observed in diabetic patients (1–3). In addition, hypertensive patients may observe vascular tortuosity due to vascular stenosis or elevated arterial blood pressure, a condition known as hypertensive retinopathy (HR) (4–7). Morphological information such as density, curvature, and thickness of retinal vessels can serve as vital signal for the diagnosis and detection of these diseases (8). To advice physicians make scientific diagnoses of these diseases, it is important to generate accurate images of retinal blood vessels of patients. However, accurately extracting retinal blood vessels is an extremely difficult challenge for the following reasons. First, retinal blood vessels vary widely in shape and size. Second, there are many complex structures and regions in retinal images, covering pathological regions, optic disc regions, hemorrhages, and exudates, which easily lead to wrong segmentation of blood vessels. Third, the weak contrast makes it difficult to distinguish vessels from the background in many edge regions. Therefore, in this task, automated algorithms and precise vessel segmentation from retinal images are in high demand, and numerous algorithms for automatic retinal vessel segmentation have been proposed (9).

Generally, retinal blood vessel segmentation algorithms can be roughly classified into two categories: unsupervised algorithms and supervised algorithms, wherein unsupervised algorithms do not provide manual annotations as reference during training. Filter-based algorithms are typical unsupervised methods. Zhang et al. (10) proposed a filter-based method, which adopts two 3D rotated frames for retinal vessel segmentation. Azzoprardi et al. (11) proposed a shift filter-response combination that can automatically detect blood vessels. Examples of other unsupervised algorithms include the method of Zhang et al. (12), which utilizes a self-organizing map for pixel clustering and further employs the Otsu algorithm to classify each neuron in the output layer as a retinal vascular neuron or a non-retinal vascular neuron. Vessel-based tracking algorithms (13) are also popular to solve the above methods. However, since the ground truth is lack, the performance of unsupervised algorithms is generally lower than that of supervised algorithms.

In recent years, deep learning models have been utilized to the field of retinal image segmentation, which shows advanced performance due to their strong data processing capabilities to capture high-level semantic features. In particular, convolutional neural networks are extensively used in numerous image processing tasks, and are also rapidly gaining traction among researchers in retinal blood vessel segmentation. Ronneberger et al. (14) proposed a well-known neural network architecture for biomedical image segmentation, called U-Net, which was originally applied to cell segmentation task and was the state-of-the-art method at that time. In addition, medical image datasets, such as retinal blood vessel image datasets, are often hard to obtain due to patient ethics and privacy concerns, resulting in the small scale of available datasets. In order to avoid overfitting, model design usually needs to pursue lightweight, and U-Net can productively enhance the performance of deep learning models in small-scale datasets. Therefore, numerous recent applications of retinal blood vessel segmentation are derived from U-Net. Fu et al. (15) improved the vessel segmentation performance by employing a model that combines the lateral output layer and a conditional random field. Zhang et al. (16) introduced AG-Net, which integrates the attention gate into the traditional guidance filter to obtain the attention guidance filter, and remove the introduced complexity noise components in the background. Wang et al. (17) proposed the DEU-Net model, in which contextual paths can capture more semantic information, and spatial paths are used to retain specific information. Zhang and Chung (18) proposed an edge-based mechanism in U-Net to achieve a bettered performance. Hu et al. (19) proposed a U-Net variant by using a saliency mechanism. Guo et al. (20) introduced Dense Residual Network (DRNet) to segment blood vessels in Scanning Laser Ophthalmoscopy (SLO) retinal images. Zhang et al. (21) proposed Pyramid U-Net, which proposed Pyramid Scale Aggregation Block (PSAB) for U-Net to aggregate multi-level features for more accurate segmentation of retinal vessels. Although the above U-Net-based methods have achieved considerable results to a certain extent, there are still the following problems. For one hand, at many peripheral regions, low contrast makes it difficult to distinguish small blood vessels from the background. For another, there are few samples used for the model, which can easily lead to overfitting problem.

To address these challenges, we propose an innovative U-Net-based network named as Bi-directional ConvLSTM Residual U-Net (BCR-UNet). The main contributions of this work are summarized as follows:

1. In order to solve the problem of overfitting caused by small samples, instead of using the data augmentation techniques, a novel Structured Dropout Residual Block (SDRB) is proposed, which introduces Dropblock regularization to enhance the robustness of the network. In this article, we replace the basic blocks of the original U-Net with SDRB to form a novel U-shaped network. In the experimental section, we explore the performance of different residual blocks to demonstrate the effectiveness of SDRB.
2. Inspired by the ability of BConvLSTM (22), we integrate BConvLSTM to the skip connections between the first residual convolutional block and the last up-convolutional layer to improve the discriminative power of the network and preserve more original semantic information of tiny blood vessels. We argue that this design is effective in handling low-contrast tiny blood vessels, and verify its effectiveness through ablation experiments.
3. Based on the above work, an innovative Bi-directional ConvLSTM Residual U-Net (BCR-UNet) is proposed to comprehensively address the challenges of retinal vessel segmentation. By comparing the segmentation results with the state-of-the-art models, the proposed BCR-UNet achieves promising performance.

## Proposed method

### Dropblock

In order to avoid the over-fitting problem of deep neural networks, a simple regularization method like Dropout is usually utilized. The main point of Dropout is that some features are randomly discarded during the training process. However, this character is effective for the fully connected layer, and it is not obvious for the convolutional layer due to the correlation between the activated cells. In other words, for the convolutional layer, even if Dropout is used, the input semantic information can still be sent to the next layer, resulting in overfitting. Intuitively, we need a structured Dropout method. Therefore, Ghiasi et al. (23) proposed Dropblock to standardize convolutional neural networks and this method has been effectively verified in SD-UNet (24). Compared with Dropout, the main difference is that Dropblock drops continuous regions in the feature map instead of randomly dropping independent units. Dropblock has two important parameters *s* and *y*, represents the size of the control discarded block, and denotes the number of active units that are discarded, which can be calculated as:


(1) $$\begin{array}{l} y=\frac{1-p}{s^{2}}\frac{f^{2}}{{\left(f-s+1\right)}^{2}} \end{array}$$

where *p* denotes the probability of keeping a certain unit active, and *f* represents the size of the feature map at that location.

### Structured dropout residual block

In the field of deep learning, residual network (ResNet) (25) is a milestone breakthrough, and has received extensive attention in the area of computer vision due to its excellent performance. In recent years, the residual module has become the basic module for many deep neural networks to be applied to the area of biomedical image segmentation (20, 26–28), and these methods achieve advanced performance. Inspired by the above methods, we also adopt the residual block as the basic unit to construct a neural network for automatically segmenting retinal vessels.

**Figure 1:**
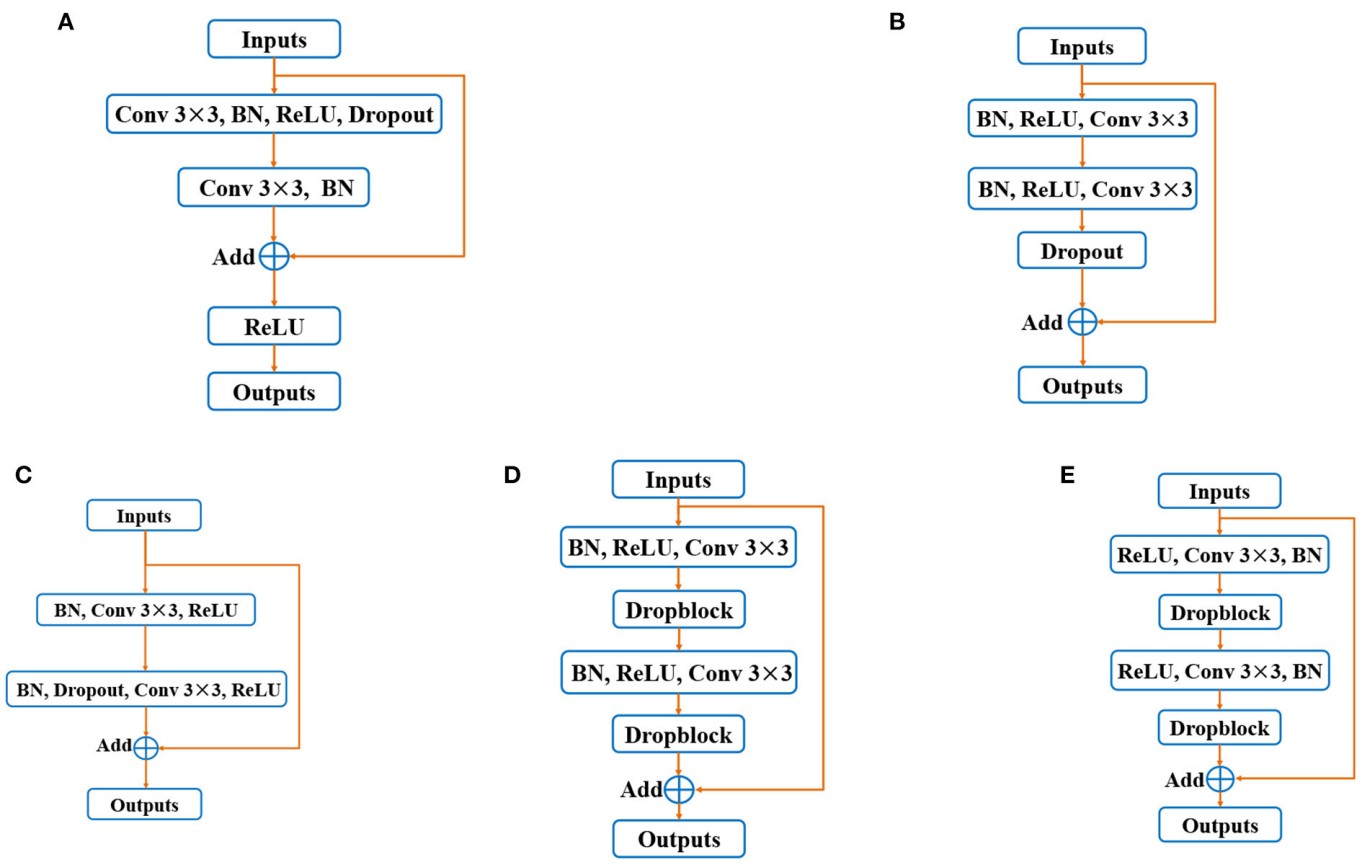
Variants of residual blocks: **(A)** Original residual block, **(B)** pre-activation residual block, **(C)** before-activation residual block, **(D)** residual block in DRNet, and **(E)** Strutured Dropout Residual Block (SDRB).

Many variants of residual blocks have been proposed in the past researches. The original residual block consists of two convolutional layers, followed by a batch normalization (BN) and ReLU layer (16) (shown in Figure 1A). In (29), He et al. introduced a new kind of residual structure named “pre-activation residual block” (see in Figure 1B). It is worth noting that this residual block achieves improved performance because it benefits from backpropagation gradient. Li et al. (30) proposed a novel residual structure named “before-activation residual block” (shown in Figure 1C), which performs better than the “pre-activation residual block,” indicating that batch normalization (BN) position plays an important role. In addition, in DRNet (20), the combination of pre-activation residual block and Dropblock brings advanced performance in retinal vessel segmentation (shown in Figure 1D). Based on the above discussion, we propose a new residual structure as the basic unit of our proposed BCR-UNet, as shown in Figure 1E, which is hereinafter referred to as the “Structured Dropout Residual Block (SDRB).” The effectiveness of SDRB has been experimentally verified and outperforms the “pre-activation residual block,” “before-activation residual block” and residual block in DRNet.

### Bi-directional ConvLSTM residual U-Net

**Figure 2:**
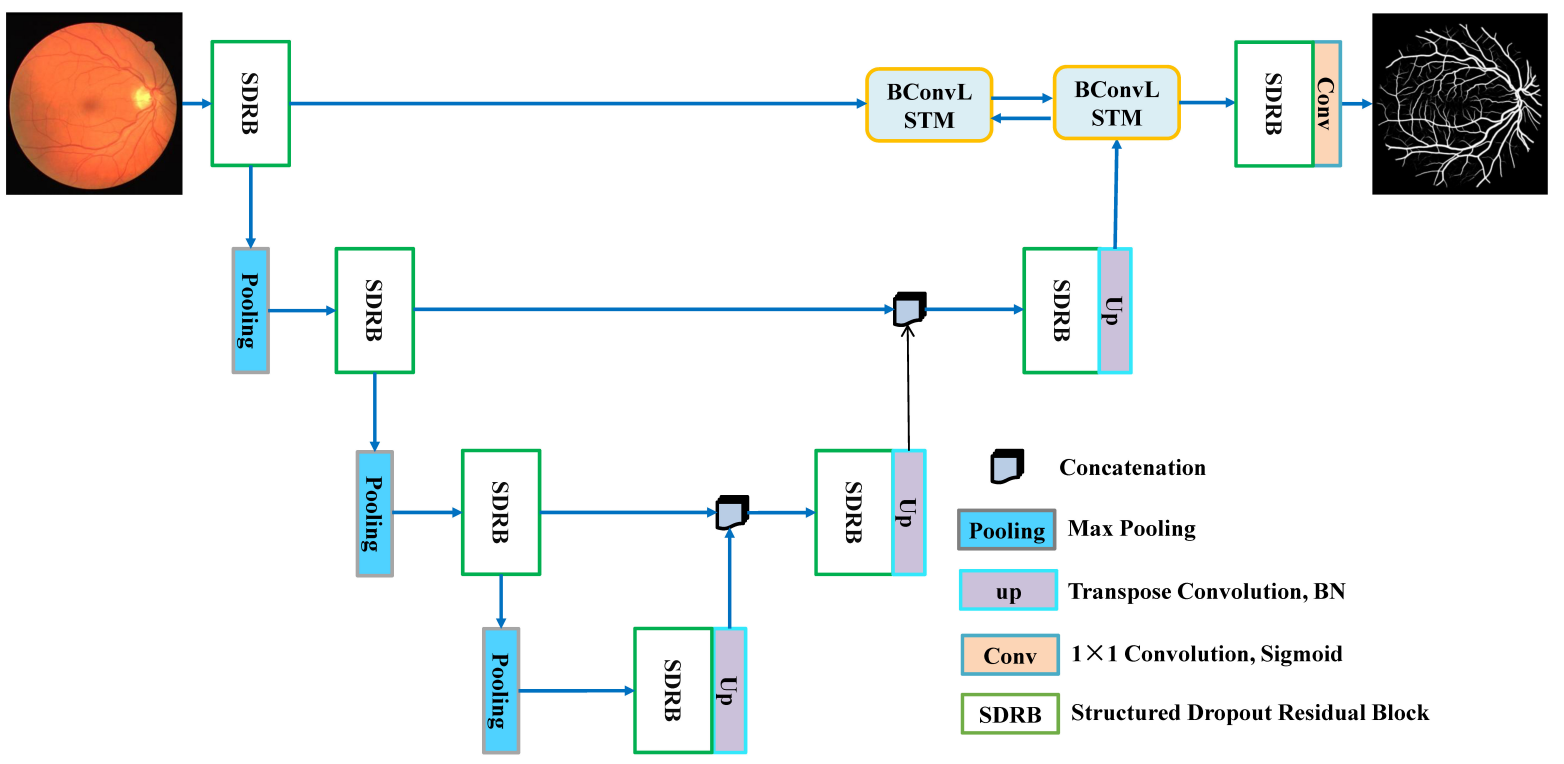
The network architecture of BCR-UNet.

**Figure 3:**
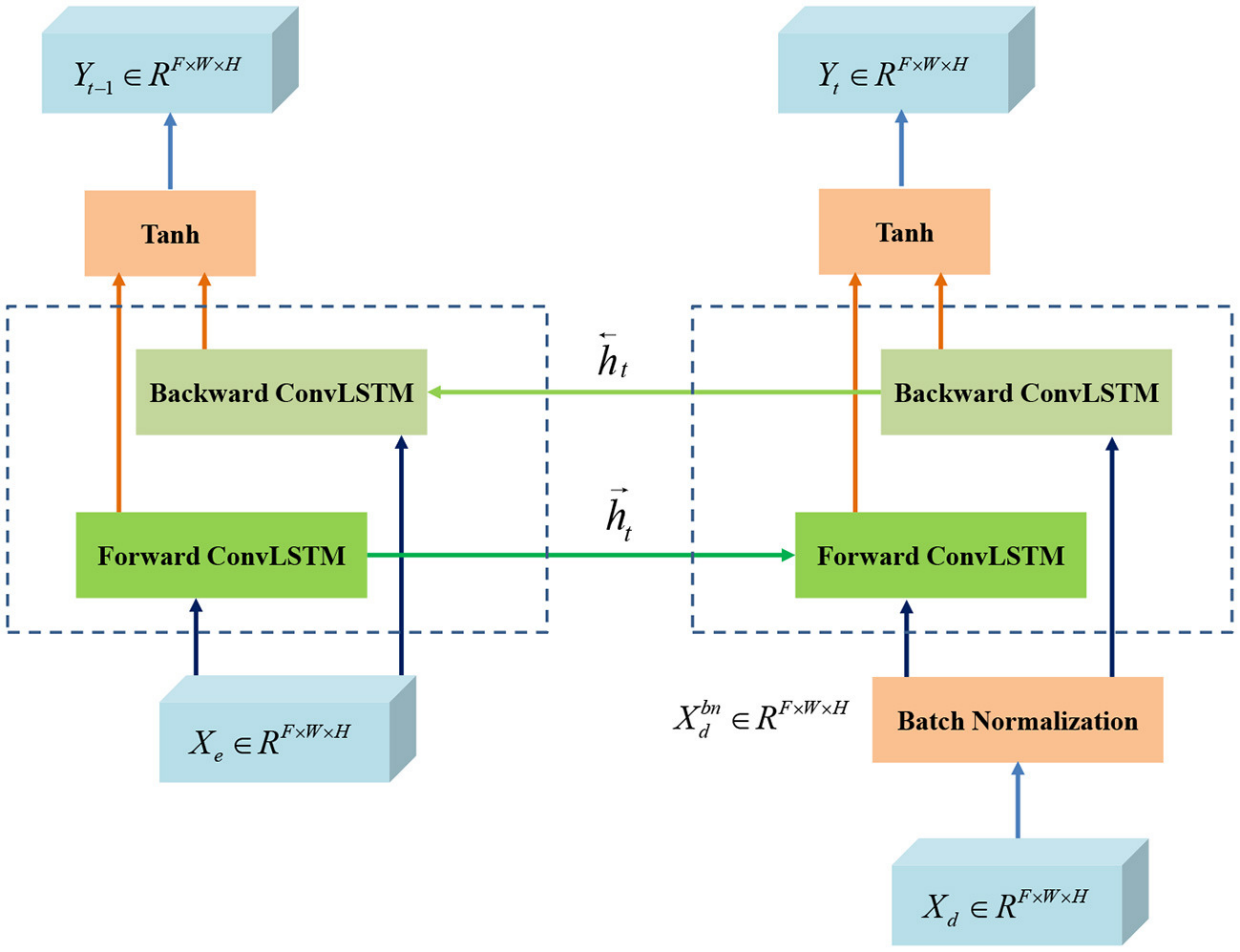
The flowchart Bi-directional ConvLSTM.

According to the design idea of UNet, BCR-UNet is primarily separated into two parts: encoder and decoder, which can realize end-to-end training. The network architecture is shown in Figure 2. The capability of the encoder is to extract a representative image feature which has a dramatic impact on the final performance of segmentation. In BCR-UNet, the encoder consists of three steps. Each step consists of a SDRB and a 2 × 2 max pooling function. The encoder captures features with high-level semantic information, and the decoder can recover the initial image information. The decoder also has three steps, and each step starts by executing an upsampling function on the output of the former step. Upsampling is performed using a transposed convolution with stride 2, followed by a BN. In the original U-Net, the matched feature maps from the encoder are replicated to the decoder, and these feature maps are then concatenated with the output of the upsampling function. Unlike U-Net, for BCR-UNet, BConvLSTM is applied to handle both feature maps in a more sophisticated manner by combining the output of the first SDRB in the encoder and the output of the last step upsampling function in the decoder. Let $X_{e}\in R^{F\times W\times H}$ be the feature maps replicated from the encoder, and $X_{d}\in R^{F\times W\times H}$be the output of the last upsampling function in the decoder, where *F* is number of feature maps, and *W*×*H* is the size of each feature map. As shown in Figure 3, *X*_*d*_ is first passed to a BN, producing $X_{d}^{bn}\in R^{F\times W\times H}$. In subsequent experiments, we verify the superior performance of this design.

### Bi-directional ConvLSTM

Standard LSTM networks utilize fully connected input-to-state and state-to-state conversions, so the primary drawback of these models is that they ignore the spatial correlations. To overcome this problem, Shi et al. (22) proposed ConvLSTM, which utilized convolution operation to input-to-state and state-to-state conversions. It is composed of an input gate *i*_*t*_, a forget gate *f*_*t*_, a memory gate *m*_*t*_, and an output gate *o*_*t*_. Input, forget and output gates act as control gates for accessing, clearing and updating the memory unit. In terms of formula, ConvLSTM can be expressed as follows:


(2) $$\begin{array}{lll} i_{t} & = & \sigma \text{(}W_{xi}⊗X_{t}+W_{hi}⊗h_{t-1}+W_{mi}⊗m_{t-1}+b_{i}\text{)}\\ f_{t} & = & \sigma \text{(}W_{xf}⊗X_{t}+W_{hf}⊗h_{t}+W_{mf}⊗m_{t-1}+b_{f}\text{)}\\ m_{t} & = & f_{t}⊙m_{t-1}+i_{t}\tanh\text{(}W_{xm}⊗X_{t}+W_{hm}⊗h_{t-1}+b_{m}\text{)}\\ o_{t} & = & \sigma \text{(}W_{xo}⊗X_{t}+W_{ho}⊗h_{t-1}+W_{mo}⊙m_{t}+b_{o}\text{)}\\ h_{t} & = & o_{t}⊙\tanh\text{(}m_{t}\text{)} \end{array}$$

where ⊗ and ⊙ represent convolution and Hadamard functions, respectively. *X*_*t*_ is the input tensor (i.e., *X*_*e*_and $X_{d}^{bn}$), *h*_*t*_ is the hidden sate tensor, *W*_*x*°_ and *W*_*h*°_ are convolution kernels corresponding to the input and hidden state, respectively, and *b*_*i*_, *b*_*f*_, *b*_*m*_ and *b*_*o*_ are the bias terms.

Although ConvLSTM is improved, it only deals with forward dependencies, which does not fully consider all the information in the sequence. Therefore, the model should consider both backward dependencies and analyze both forward and backward dependencies to improve forecasting accuracy (31). BConvLSTM employs two ConvLSTMs to deal with the input data into both forward and backward directions, and then makes decisions for the current input by processing the data dependencies in the two directions. Therefore, in this work, we utilize BConvLSTM (22) to encode *X*_*e*_ and $X_{d}^{bn}$. The output formula of BConvLSTM is:


(3) $$\begin{array}{l} Y_{t}=\tanh\;\left({\text{W}}_{y}^{\vec{h}}⊗\vec{h}+{\text{W}}_{y}^{\overset{\leftarrow }{h}}⊗\overset{\leftarrow }{h}+b\right) \end{array}$$

where ${\vec{h}}_{t}$ indicates the hidden state tensors for forward states, while ${\overset{\leftarrow }{h}}_{t}$for backward states, *b* is the bias term, and $Y_{t}\in R^{F\times W\times H}$ represents the final output considering two-way spatiotemporal information. In addition, Tanh stands for hyperbolic tangent, which is used to combine the outputs of the forward and backward states in a non-linear fashion.

## Experiments and results

### Materials and implementation details

To evaluate the performance of BCR-UNet, we select four publicly available retinal image datasets, including DRIVE (32), CHASE DB1 (33), STARE (34) and IOSTAR (10), whose specific information can be found in Table 1. In addition, in order to quantitatively evaluate the performance of BCR-UNet, we choose accuracy (*ACC*), Sensitivity (*SEN*), specificity (*SPE*), *F*1-score (*F*1), the *area under the curve* (*AUC*) of the receiver operating characteristic curve (ROC), *Intersection-over-Union* (*IOU*) and *Matthews correlation coefficient* (*MCC*) as evaluation metrics. These metrics are defined as follows:


(4) $$\begin{array}{lll} SEN & = & \frac{Tp}{Tp+Fn} \end{array}$$

(5) $$\begin{array}{lll} SPE & = & \frac{Tn}{Tn+Fp} \end{array}$$

(6) $$\begin{array}{lll} ACC & = & \frac{Tp+Tn}{Tp+Tn+Fp+Fn} \end{array}$$

(7) $$\begin{array}{lll} IOU & = & \frac{Tp}{Fp+Tp+Fn} \end{array}$$

(8) $$\begin{array}{lll} F1 & = & \frac{2Tp}{2Tp+Fp+Fn} \end{array}$$

(9) $$\begin{array}{lll} MCC & = & \frac{Tp\times Tn-Fp\times Fn}{\sqrt{\text{(}Tp+Fp\text{)}\times \text{(}Tp+Fn\text{)}\times \text{(}Tn+Fp\text{)}\times \text{(}Tn+Fn\text{)}}} \end{array}$$

**Table 1:** The detail information of four datasets.

**Datasets**	**Source**	**Count**	**Train/Test**	**Resolution**
DRIVE	Dutch Diabetic Retinopathy Screening Program	40	20/20	565 × 584
CHASE DB1	Children's Heart and Health Study in England	28	20/8	999 × 960
STARE	Structural Analysis of the Retina	20	15/5 (4-fold cross-validation)	700 × 605
IOSTAR	EasyScan Camera (i-Optics Inc., Netherlands)	30	20/10	1024 × 1024

where Tp denotes as true positive, which means that when a predicted pixel is compared with a pixel at the same position in the ground truth value, the predicted pixel is accurately classified as a blood vessel. Tn denotes as true negative, which denotes that when a predicted pixel is compared with a pixel at the same position in the ground truth value, then the predicted pixel is correctly divided as a non-vascular. Correspondingly, Fp is a false positive value, which represents that one of the pixels is classified as a blood vessel in the segmented image, and the corresponding pixel with the same position in the ground truth image is a non-vascular pixel. Fn is defended as a false negative value, which means that one of the pixels is classified as non-vascular in the predicted image, and the corresponding pixel with the same position in the ground real image is the vascular pixel. In addition, ACC is an area under the receiver operating characteristic curve (ROC), which measures the segmentation performance based on recall and precision, and is not affected by imbalanced data such as retinal blood vessel images. IOU is a number that evaluates the degree of overlap between two regions (i.e., group truth and detection region). F1 is defined as a weighted mean of precision and recall, where precision denotes as the number of Tp divided by sum of Tp and Fp, while recall defines as the number of Tp divided by the total number of Tp and Fp. MCC is a very effective evaluation metric, which often used to test the performance of a classification model under the two classes are imbalance case.

The implementation of our proposed BCR-UNet is based on Keras with Tensorflow as the backend and a Tesla V100 graphics card with 32GB of memory. For the training images of the four datasets, we adopt random horizontal, rotation and diagonal and vertical flips for augmentation, and randomly select 10% of the augmented images as the validation set. In training phase, we employ Adam with a learning rate of 0.001 as the optimization method and binary cross-entropy as the loss function. In our experiments, the batch size is set to 2, except for the STARE dataset, which is trained for 300 epochs, and the other datasets are trained for 100 epochs. In addition, for the setting of Dropblock, we uniformly set the drop block size to 7 and the dropout rate is 0.2.

### Ablation studies

**Figure 4:**
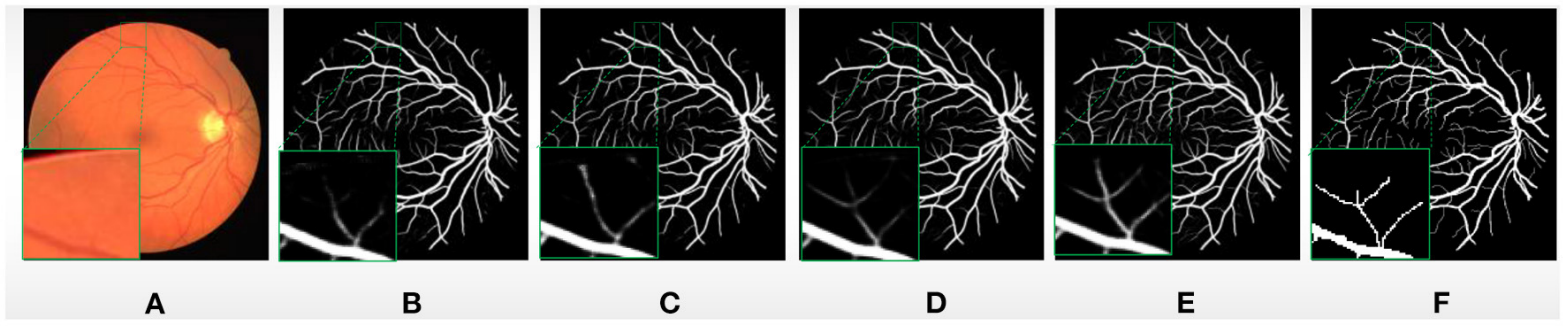
**(A)** A typical image from DRIVE dataset, **(B)** Baseline, **(C)** Baseline+BConvLSTM, **(D)** Baseline+Dropblock, **(E)** BCR-UNet, and **(F)** ground truth.

In order to verify the effectiveness of our proposed BCR-UNet model, we conduct ablation studies to prove the effectiveness of each component in the first experiment. As mentioned before, the Structured Dropout Residual Block (SDRB) includes Dropblock. In order to be able to verify the effectiveness of Dropblock, SDRBs without Dropblock is used to construct a U-shaped network (i.e., BCR-UNet w/o Dropblock and Bi-ConvLSTM) and treat the obtained model as Baseline. Table 2 shows the segmentation performance of Baseline, Baseline+BConvLSTM, Baseline+Dropblock and BCR-UNet (i.e., Baseline+Dropblock+BConvLSTM) from top to bottom, respectively. The visual effects of different components are shown Figure 4.

**Table 2:** The ablation experiments on four datasets.

**Models**	***ACC***	***SEN***	***SPE***	***AUC***	***F1***	***IOU***	***MCC***
**DRIVE**							
Baseline	0.9681	0.7595	0.9881	0.9834	0.8065	0.6757	0.7910
Baseline+BConvLSTM	0.9681	0.7694	**0.9872**	0.9827	0.8084	0.6784	0.7922
Baseline+Dropblock	0.9693	0.7841	0.9870	0.9860	0.8171	0.6908	0.8012
BCR-UNet	**0.9695**	**0.8183**	0.9840	**0.9866**	**0.8246**	**0.7015**	**0.8075**
**CHASE DB1**							
Baseline	0.9733	0.8253	0.9833	0.9867	0.7958	0.6609	0.7821
Baseline+BConvLSTM	0.9739	0.8115	0.9848	0.9869	0.7966	0.6619	0.7828
Baseline+Dropblock	0.9754	0.8327	**0.9850**	0.9891	0.8101	0.6808	0.7973
BCR-UNet	**0.9755**	**0.8383**	0.9847	**0.9898**	**0.8118**	**0.6832**	**0.7992**
**STARE**							
Baseline	0.9702	0.7647	0.9870	0.9746	0.7948	0.6602	0.7800
Baseline+BConvLSTM	0.9706	0.7673	0.9872	0.9791	0.7975	0.6640	0.7832
Baseline+Dropblock	0.9742	0.8006	**0.9883**	**0.9885**	0.8238	0.7010	0.8115
BCR-UNet	**0.9743**	**0.8308**	0.9860	0.9873	**0.8302**	**0.7103**	**0.8168**
**IOSTAR**							
Baseline	0.9709	0.7415	**0.9909**	0.9870	0.8030	0.6708	0.7905
Baseline+BConvLSTM	0.9705	0.7624	0.9886	0.9860	0.8055	0.6743	0.7911
Baseline+Dropblock	0.9706	0.7793	0.9885	**0.9884**	0.8152	0.6879	0.8009
BCR-UNet	**0.9727**	**0.7965**	0.9880	0.9882	**0.8234**	**0.6999**	**0.8091**

Bold values is the highest scores for the metrics.

#### Effectiveness of BConvLSTM

First, we just add the BConvLSTM module to the Baseline (i.e., Baseline+ BConvLSTM) and apply it to the DRIVE, CHASE DB1, STARE, and IOSTAR datasets. A typical example of retinal blood vessel segmentation results in DRIVE is shown Figure 4. This experiment results obviously shows that the using BConvLSTM module can productively segment blood vessels of various scales, especially some small blood vessels that Baseline cannot handle well. As shown in Table 2, compared with both Baseline, Baseline+BConvLSTM improves the performance from 67.57% / 66.09% / 66.02% / 67.08% to 67.84% / 66.19% / 66.40% / 67.43% in terms of *IOU*, and for *MCC*, the performance is improved from 79.10% / 78.21% / 78% / 79.05% to 79.22% / 78.28% / 78.32% / 79.11%. Further, we evaluate the effect of BConvLSTM by comparing the performance of Baseline+Dropblock and BCR-UNet (i.e., Baseline+Dropblock+ BConvLSTM) on each dataset. Compared with Baseline + Dropblock, we can notice that for *IOU*, the performance of BCR-UNet has improved by 1.07% / 0.23% / 0.93% / 1.2%, for *MCC*, the performance is improved by 0.63% / 0.19% / 0.53% / 0.82% and for other metrics, there are increases to some extent. Therefore, our experimental results and segmentation results clearly prove the importance of BConvLSTM in the application.

#### Effectiveness of dropblock

In this subsection, we investigate the effectiveness of the Dropblock. The results of different methods on the four datasets, as shown in Table 2, compared with the Baseline, the introduced Dropblock module (i.e., Baseline+Dropblock) increases *IOU* by 1.51% / 2% / 4.08% / 1.71% (from 67.57% / 66.09% / 66.02% / 67.08% to 69.08% / 68.09% / 70.1% / 68.79%), and *MCC* has increased 1.02% / 1.52% / 3.15% / 1.04% (from 79.10% / 78.21% / 78% / 79.05% to 80.12% / 79.73% / 81.15% / 80.09%). For *F*1, *AUC* and other indicators have also been improved due to the addition of Dropblock. In addition, to verify the superiority of Dropblock, we add Baseline+Dropout experiments, and the results of Baseline+Dropblock and Baseline+Dropout on four datasets are shown in Table 3. The results present that Dropblock is better than Dropout in all comprehensive metrics in all datasets, which demonstrates that Dropblock is obviously effective in this work. BCR-UNet incorporates Dropblock and BConvLSTM into the Baseline (i.e., Baseline+ Dropblock+BConvLSTM) to evaluate the complementarily between the two modules. As shown in Table 2, the segmentation accuracy has been greatly improved, in *IOU*, there is a significant increase of about 2.58% / 2.23% / 5.01% / 2.91%, and for *MCC* it is increased by about 1.65% / 1.71% / 3.68% / 1.86%, which is enough to show that the combination of Dropblock and BConvLSTM in our BCR-UNet is effective.

**Table 3:** Comparative experiments of Dropblock and Dropout on four datasets.

**Datasets**	**Models**	***AUC***	***F1***	***IOU***	***MCC***
DRIVE	Baseline+Dropout	0.9841	0.8147	0.6873	0.7994
	Baseline+Dropblock	**0.9860**	**0.8171**	**0.6908**	**0.8012**
	U-Net	0.9849	0.8170	0.6907	0.8007
CHASE DB1	Baseline+Dropout	0.9848	0.7976	0.6634	0.7844
	Baseline+Dropblock	**0.9892**	**0.8101**	**0.6809**	**0.7973**
	U-Net	0.9873	0.7989	0.6652	0.7853
STARE	Baseline+Dropout	0.9802	0.8076	0.6781	0.7945
	Baseline+Dropblock	**0.9885**	**0.8238**	**0.7010**	**0.8115**
	U-Net	0.9813	0.8026	0.6709	0.7887
IOSTAR	Baseline+Dropout	0.9874	0.8019	0.6694	0.7886
	Baseline+Dropblock	**0.9884**	**0.8152**	**0.6879**	**0.8009**
	U-Net	0.9873	0.8104	0.6813	0.7967

Bold values is the highest scores for the metrics.

### Effectiveness of SDRB

**Figure 5:**
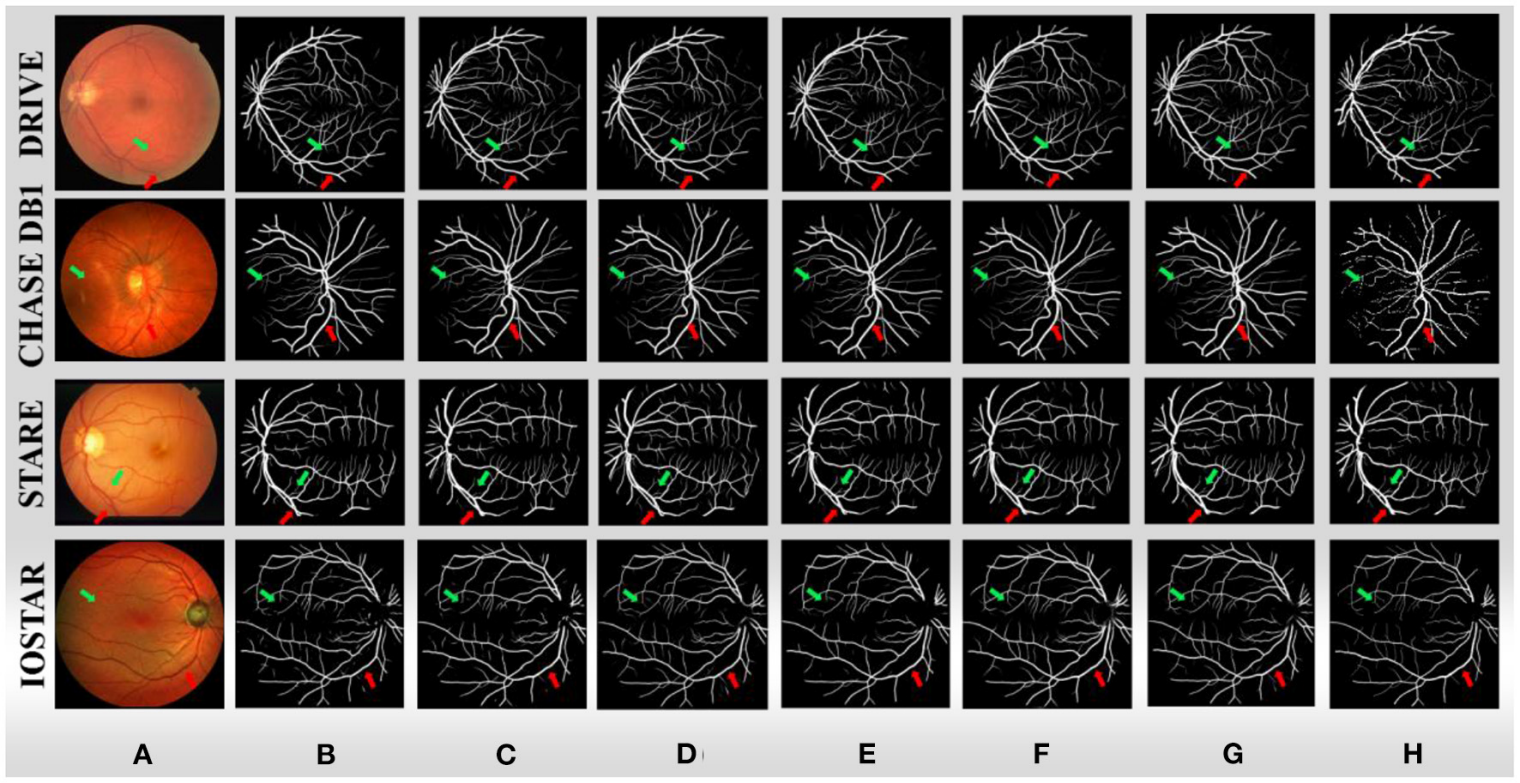
**(A)** Sample images from four datasets, **(B)** U-Net, **(C)** Attention UNet, **(D)** SD-UNet, **(E)** MultiResUNet, **(F)** DRNet, **(G)** BCR-UNet, and **(H)** ground truths.

In order to verify that the proposed SDRB is meaningful in the application of retinal blood vessel segmentation, we add the segmentation performance of U-Net to Table 3. Compared with U-Net, the performance of Baseline+Droblock (i.e., the model built with SDRBs) is better than U-Net in all indicators. In addition, we conduct several experiments to study the segmentation effect in different residual blocks. Specifically, we consider the following variants of the residual block: (1) the raw residual block (Figure 1A), (2) the pre-activated residual block (Figure 1B), (3) the before activation residual block (Figure 1C), (4) the modified residual block comes from DRNet (Figure 1D), (5) the proposed SDRB (Figure 1E). We conduct experiments by integrating the above blocks into Baseline. In short, these residual block variants replace the basic residual block of Baseline. For ease of reference, we refer to these five U-shaped networks as RUNet_x, where x represents the subgraph number of Figure 1, that is, RUNet_a is a residual network constructed using the original residual block in Figure 1, and so on. We report the results on the DRIVE dataset, the highest scores for the metrics in Table 4 are shown in bold, and the results show that RUNet_e (i.e., Baseline+Dropblock) performs the best. The above discussion and the results from Tables 3, 4 show that SDRB is effective for constructing novel U-shaped networks.

**Table 4:** Comparative experiments of different residual blocks on DRIVE dataset.

**Models**	***ACC***	***SEN***	***SPE***	***AUC***	***F1***	***IOU***	***MCC***
RUNet_a	0.9680	0.8091	0.9833	0.9831	0.8159	0.6891	0.7984
RUNet_b	0.9686	0.7988	0.9850	0.9826	0.8170	0.6906	0.8001
RUNet_c	0.9676	**0.8246**	0.9814	0.9836	0.8170	0.6906	0.7993
RUNet_d	0.9678	0.8219	0.9818	0.9853	**0.8173**	**0.6911**	0.7997
RUNet_e (i.e. Baseline+Dropblock)	**0.9693**	0.7841	**0.9870**	**0.9860**	0.8171	0.6908	**0.8012**

Bold values is the highest scores for the metrics.

### Comparison with state-of-the-art models

We further compare the performance of BCR-Net with multiple state-of-the-art and widely used methods. As shown in Tables 5, 6, M-Net (35), AG-Net (16), RSAN (36), NFN+(37), Pyramid U-Net (21), SCS-Net (38), Deng and Ye (39) and Xu et al. (40) gave the experimental results of DRIVE and CHASE DB1 in the original paper, and also gave STARE and IOSTAR in part. For the other five methods, including U-Net (5), Attention UNet (41), SD-UNet (24), MultiResUNet (27) and DRNet (20), we conduct experiments on four datasets (DRIVE, CHASEDB1, STARE, and IOSTAR) based on the same training strategy and parameter settings as BCR-UNet. Quantitatively, as shown in Tables 5–8, our proposed BCR-UNet achieves the highest *AUC* of 0.9866 / 0.9898 / 0.9873 / 0.9882, the highest *F*1 of 0.8246 / 0.8118 / 0.8302 / 0.8234, the highest *IOU* of 0.7015 / 0.6832 / 0.7103 / 0.6999, and the highest *MCC* of 0.8075 / 0.7992 / 0.8168 / 0.8091 on the four datasets, while other three metrics are also comparable. From the perspective of segmentation visual effects, the segmentation results of BCR-UNet and other competing methods in four datasets are shown in Figure 5. For four samples from four datasets, it is clear that BCR-UNet can predict most of the thick and tiny vessels (indicated by red and green arrows) compared to other competing models. As a general benchmark for medical image segmentation, U-Net performs poorly in this task because many peripheral blood vessels are not accurately segmented. Although Attention U-Net introduces an attention mechanism, it does not show superiority in this work compared to U-Net. The performance of SD-UNet is improved due to the introduction of Dropblock, but limited by the benchmark network itself, it cannot adapt well to complex vessel trees, especially some vessel intersection regions. MultiResUNet employs the residual convolution mechanism to improve the performance to a certain extent, and the effect is better than U-Net, but the robustness is relatively poor, because the performance is only better than SD-UNet on the DRIVE and STARE datasets. DRNet performs well on the IOSTAR dataset, confirming that it is more suitable for segmenting blood vessels in Scanning Laser Ophthalmoscopy (SLO) retinal images, but fails to preserve enough tiny vessels on the other three datasets. For our proposed BCR-UNet, the tiny blood vessels at the vessel terminals can be accurately segmented on all four datasets, as indicated by the green arrows. Overall, our BCR-UNet network generally outperforms other state-of-the-art models because the combination of SDRB, BConvLSTM modules makes the network more robust and can effectively preserve tiny vessels at low-contrast vessel-end regions.

**Table 5:** Results of BCR-UNet and other methods on DRIVE dataset.

**Models**	***ACC***	***SEN***	***SPE***	***AUC***	***F1***	***IOU***	***MCC***
M-Net (35)	0.9674	0.7680	0.9868	0.9829	-	0.6726	-
AG-UNet (16)	0.9692	0.8100	0.9848	0.9856	-	0.6965	-
RSAN (36)	0.9691	0.8149	0.9839	0.9855	0.8222	-	-
NFN+ (37)	0.9668	0.8002	0.9790	0.9832	-	-	-
Pyramid U-Net (21)	0.9615	0.8213	0.9807	0.9815	-	-	-
SCS-Net (38)	0.9697	0.8289	0.9838	0.9837	-	-	-
Deng et al. (39)	0.9683	**0.8363**	0.9811	-	0.8211	-	-
Xu et al. (40)	0.9689	0.8342	0.9821	0.9858	-	-	-
U-Net (5)	0.9690	0.7906	0.9861	0.9849	0.8170	0.6907	0.8007
Attention UNet (41)	0.9685	0.7663	**0.9879**	0.9834	0.8099	0.6805	0.7943
SD-UNet (24)	0.9695	0.7831	0.9874	0.9854	0.8182	0.6923	0.8025
MultiResUNet (27)	**0.9697**	0.7825	0.9876	0.9859	0.8188	0.6931	0.8033
DRNet (20)	0.9672	0.7967	0.9836	0.9815	0.8099	0.6804	0.7921
BCR-UNet	0.9695	0.8183	0.9840	**0.9866**	**0.8246**	**0.7015**	**0.8075**

Bold values is the highest scores for the metrics.

**Table 6:** Results of BCR-UNet and other methods on CHASE DB1 dataset.

**Models**	***ACC***	***SEN***	***SPE***	***AUC***	***F1***	***IOU***	***MCC***
M-Net (35)	0.9729	0.7922	0.9851	0.9845	-	0.6483	-
AG-UNet (16)	0.9743	0.8186	0.9848	0.9863	-	0.6669	-
RSAN (36)	0.9751	0.8486	0.9836	0.9894	0.8111	-	-
NFN+ (37)	0.9735	0.7933	0.9855	0.9832	-	-	-
Pyramid U-Net (21)	0.9639	0.8035	0.9787	0.9832	-	-	-
SCS-Net (38)	0.9744	0.8365	0.9839	0.9867	-	-	-
Deng et al. (39)	0.9714	0.8541	0.9794	-	-	-	0.7900
Xu et al. (40)	0.9749	0.8477	0.9837	0.9881	-	-	-
U-Net (5)	0.9744	0.8074	0.9856	0.9873	0.7989	0.6652	0.7853
Attention UNet (26)	0.9750	0.8185	0.9856	0.9891	0.8053	0.6740	0.7921
SD-UNet (24)	**0.9756**	0.8167	**0.9863**	0.9893	0.8085	0.6786	0.7955
MultiResUNet (27)	0.9755	0.8178	0.9861	0.9891	0.8082	0.6781	0.7952
DRNet (20)	0.9755	0.8298	0.9853	0.9897	0.8100	0.6806	0.7971
BCR-UNet	0.9755	**0.8383**	0.9847	**0.9898**	**0.8118**	**0.6832**	**0.7992**

Bold values is the highest scores for the metrics.

**Table 7:** Results of BCR-UNet and other methods on STARE dataset.

**Models**	***ACC***	***SEN***	***SPE***	***AUC***	***F1***	***IOU***	***MCC***
NFN+ (37)	0.9727	0.8096	0.9843	0.9844	-	-	-
SCS-Net (38)	0.9736	0.8207	0.9839	0.9877	-	-	-
Deng et al. (39)	0.9732	0.8272	0.9847	-	0.8196	-	-
U-Net (5)	0.9713	0.7726	0.9876	0.9813	0.8026	0.6709	0.7887
Attention UNet (41)	0.9718	0.7553	**0.9896**	0.9807	0.8008	0.6687	0.7881
SD-UNet (24)	0.9719	0.7913	0.9865	0.9816	0.8094	0.6806	0.7957
MultiResUNet (27)	0.9730	0.7837	0.9883	0.9730	0.8137	0.6870	0.8017
DRNet (20)	0.9724	0.7855	0.9878	0.9805	0.8110	0.6830	0.7975
BCR-UNet	**0.9743**	**0.8308**	0.9860	**0.9873**	**0.8302**	**0.7103**	**0.8168**

Bold values is the highest scores for the metrics.

**Table 8:** Results of BCR-UNet and other methods on IOSTAR dataset.

**Models**	***ACC***	***SEN***	***SPE***	***AUC***	***F1***	***IOU***	***MCC***
NFN+ (37)	0.9683	0.7921	0.9812	0.9803	-	-	-
SCS-Net (38)	0.9706	0.8255	0.9830	0.9865	-	-	-
U-Net (5)	0.9714	0.7642	**0.9894**	0.9873	0.8104	0.6813	0.7967
Attention UNet (41)	0.9701	0.7711	0.9874	0.9865	0.8049	0.6735	0.7896
SD-UNet (24)	0.9717	0.7835	0.9881	0.9880	0.8159	0.6890	0.8014
MultiResUNet (27)	0.9712	0.7795	0.9879	0.9832	0.8125	0.6842	0.7978
DRNet (20)	0.9717	0.8191	0.9850	0.9880	0.8223	0.6983	0.8070
BCR-UNet	**0.9727**	**0.7965**	**0.9880**	**0.9882**	**0.8234**	**0.6999**	**0.8091**

Bold values is the highest scores for the metrics.

## Conclusions

U-Net is a neural network widely used in medical image segmentation. But for specific tasks such as retinal vessel segmentation, the original U-Net may not be the most suitable. Therefore, in this paper, we propose a novel U-shaped network, Bi-directional ConvLSTM Residual U-Net (BCR-UNet), for accurate segmentation of blood vessels in retinal images. In BCR-UNet, we propose a different residual block, which changes the position of BN and ReLU compared with the original residual block, and introduces Dropblock to replace Dropout to better alleviate the overfitting problem. Structued Dropout Residual Block (SDRB) is designed and is used as the basic block to build a new U-shaped network. In addition, we introduced BConvLSTM and applied it to the skip connection between the first residual block and the last residual block to improve the discriminative ability of the network. We evaluate the proposed BCR-UNet on four publicly available retinal image datasets, which are DRIVE, CHASE DB1, STARE and IOSTAR. Through ablation experiments, we verify the effectiveness of each module of BCR-UNet and by comparing with some other commonly used and state-of-the-art segmentation models. BCR-UNet has the best performance on all four datasets, indicating that BCR-UNet achieves the state-of-the-art performance. In the later research, we will conduct in-depth research on multi-task learning/cross-domain learning for solving the small sample problem in the field of medical image processing.

## Data availability statement

The source codes of the proposed network are available from the corresponding author upon request. The data are derived from public domain resources and the download links are given below: DRIVE: https://drive.grand-challenge.org/ CHASE DB1: https://researchdata.kingston.ac.uk/96/ STARE: https://cecas.clemson.edu/~ahoover/stare/ IOSTAR: http://www.retinacheck.org/download-iostar-retinal-vessel-segmentation-dataset.

## Author contributions

YY and CG: data curation, funding acquisition, methodology, supervision, writing—original draft, and writing—review and editing. WZ and WW: data curation and methodology. YH and CG: data curation, formal analysis, supervision, and writing—review and editing. All authors contributed to the article and approved the submitted version.

## Funding

This work was supported in part by grants from the National Natural Science Foundation of China (Nos. 62062040, 62102270, 61967010, and 62067003), the Outstanding Youth Project of Jiangxi Natural Science Foundation (No. 20212ACB212003), the Jiangxi Province Key Subject Academic and Technical Leader Funding Project (No. 20212BCJ23017), the National Natural Science Foundation of Liaoning Province (No. 2020-MS-239), the Key scientific research projects of Liaoning Provincial Department of Education (No. LZD202002), and the Teaching Reform Project of Colleges and Universities in Jiangxi Province (JXJG-19-2-24).

## Conflict of interest

The authors declare that the research was conducted in the absence of any commercial or financial relationships that could be construed as a potential conflict of interest.

## Publisher's note

All claims expressed in this article are solely those of the authors and do not necessarily represent those of their affiliated organizations, or those of the publisher, the editors and the reviewers. Any product that may be evaluated in this article, or claim that may be made by its manufacturer, is not guaranteed or endorsed by the publisher.
